# ALKBH5 Reduces BMP15 mRNA Stability and Regulates Bovine Puberty Initiation Through an m6A-Dependent Pathway

**DOI:** 10.3390/ijms252111605

**Published:** 2024-10-29

**Authors:** Xiaorui Yang, Ziming Wang, Yue Chen, He Ding, Yi Fang, Xin Ma, Hongyu Liu, Jing Guo, Jing Zhao, Jun Wang, Wenfa Lu

**Affiliations:** 1 Key Laboratory of Animal Production, Product Quality and Security, Ministry of Education, Jilin Agricultural University, Changchun 130118, China; yangxiaorui2020@163.com (X.Y.); wangzm0613@163.com (Z.W.); cy463374734@163.com (Y.C.); dinghe1103@126.com (H.D.); fangyi@iga.ac.cn (Y.F.); maxin3202@163.com (X.M.); jlndlhy0133@163.com (H.L.); jgchn@163.com (J.G.); jlndzjing@126.com (J.Z.); 2 Jilin Province Engineering Laboratory for Ruminant Reproductive Biotechnology and Healthy Production, College of Animal Science and Technology, Jilin Agricultural University, Changchun 130118, China

**Keywords:** bovine, m6A methylation, puberty, HPO axis, ovarian granulosa cells

## Abstract

The timing of puberty significantly influences subsequent reproductive performance in cattle. N6-methyladenosine (m6A) is a key epigenetic modification involved in the regulation of pubertal onset. However, limited research has investigated alterations in m6A methylation within the hypothalamic–pituitary–ovarian (HPO) axis during the onset of puberty. In this study, combined analysis of methylated RNA immunoprecipitation sequencing (MeRIP-Seq) and RNA sequencing (RNA-seq) is used to describe the overall modification pattern of m6A in the HPO axis, while GSEA, KEGG, and GO analyses are used to describe the enrichment pathways of differentially expressed genes and differentially methylated genes. The m6A modifications of the differential genes KL, IGSF10, PAPPA2, and BMP15 and the pathways of cell adhesion molecules (CAMs), TGF-β, cell cycle, and steroid hormone synthesis may play roles in regulating the function of the HPO axis tissue during pubertal transition. Notably, BMP15′s m6A modification depends on the action of the demethylase ALKBH5, which is recognized by the reader protein YTHDF2, promoting bovine granulosa cell proliferation, steroid production, and estrogen secretion. This study reveals for the first time the modification mechanism of BMP15 m6A during the initiation of bovine puberty, which will provide useful information for improving the reproductive efficiency of Chinese beef cattle.

## 1. Introduction

The hypothalamic–pituitary–ovarian (HPO) axis plays a pivotal role in controlling estrus activity in mammals, with the onset of puberty being influenced by a combination of genetic and environmental factors [1]. The timing of puberty onset is critical for the lifetime reproductive performance of animals [2]. High-throughput sequencing technologies have enabled the identification of several genes associated with puberty initiation in various components of the HPO axis. For instance, in the hypothalamus and ovary of goats, genes such as TCF12, TEAD4, STAT1, STAT2, GATA3, CNTN2, and THBS1 have been implicated in regulating the onset of puberty [3,4]. In Brahmin heifers, five differentially expressed transcription factors have been identified in the hypothalamus [5], and 48 genes associated with puberty have been found in the pituitary and ovary, respectively [6]. To date, however, most studies have focused on individual tissues of the HPO axis or associations between two tissues, and there is a paucity of research examining the entire HPO axis during the onset of puberty.

In addition to genetic determinants, the timing of puberty onset is influenced by environmental factors. Even within the same breed, variations in the onset of puberty can be observed due to differences in the rearing environment [7]. These variations may be primarily attributed to epigenetic modifications that alter gene expression patterns and networks, leading to context-dependent changes in reproductive function [8]. Alternative splicing (AS) and differentially expressed AS (DEAS) events in the HPO axes of gilts have been shown to be primarily enriched in pathways such as thyroid hormone signaling, oxytocin signaling, oocyte meiosis, and GnRH signaling [9]. CircRNAs produced by the AS of genes such as ESR1, JAK2, NF1, and ARNT in the sow’s ovaries may also regulate ovarian function at the onset of puberty [10].

N6-methyladenosine (m6A) modifications are the most common form of RNA modification in eukaryotic mRNA, typically occurring on RACH-consistent sequences (R = purine, A or G; H = non-G base, A, C, or U) located near stop codons or 3′ untranslated regions (UTRs) [11]. Previous studies have shown that the demethylase FTO in granulosa cells can delay FOS-dependent ovarian aging [12]; m6A can ensure the stability of mRNA in oocytes and the decay of dual cell specific transcripts after fertilization in a specific context during the transition from mother to zygote [13]; m6A regulates the ovarian estrous cycle in yaks [14], but the m6A marker genes in pre- and post-pubertal bovine tissues across the HPO axis have not yet been extensively studied.

In this study, we collected the hypothalamus, pituitary, and ovaries of bovines before and after puberty and used MeRIP-seq technology to obtain the whole transcriptome map of m6A. By analyzing the distribution of various m6A peaks across these HPO tissues, we investigated the expression patterns of m6A-modified genes within the HPO axis. Specifically, we investigated the effect of BMP15, an ovarian m6A modification gene, on estrogen secretion and the proliferation of granulosa cells through the action of the demethylase ALKBH5 in bovine ovarian granulosa cells. This study provides new insights into the mechanisms that control the onset of puberty in female mammals, particularly those involving m6A methylation modifications.

## 2. Results

### 2.1. HPO Axis-Sequencing Quality Control and Identification of Peaks Before and After Puberty

**Figure 1:**
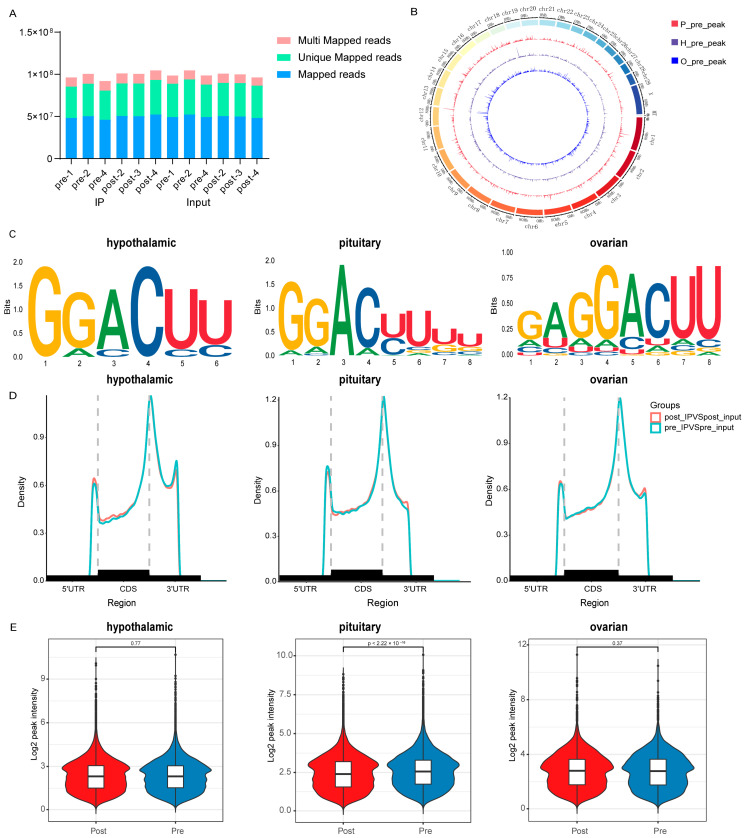
HPO axis-sequencing quality control and identification of peaks before and after puberty. (**A**) Distribution of various read types across each sample. (**B**) Distribution of m6A peak sites on the bovine genome from samples collected before first puberty. The outermost circle displays the chromosomal distribution, while the red, purple, and blue circles represent the pituitary gland, hypothalamus, and ovary, respectively. (**C**) Motif analysis using HOMER reveals the presence of the m6A motif in bovine hypothalamus, pituitary, and ovarian samples. (**D**) Density distribution of m6A peaks in hypothalamus, pituitary, and ovary transcripts before and after first puberty under different gene structures. (**E**) Global m6A levels in bovine hypothalamus, pituitary, and ovarian samples before and after first puberty.

After comparing the results of mRNA seq and MeRIP seq with the reference genome bos taurus (Version: v107), it was found that due to the enrichment of methylated fragments by m6A antibodies, there may be a high enrichment of m6A modified segments in the genome. The results showed that approximately 96–98% of the reads could match the genome of which 73–78% matched uniquely at one location in the genome, and 19–24% matched at multiple locations (Figure 1A and Supplementary Tables S1–S3). Based on the reference genome region information, valid data that matched the reference genome could be defined as matching exons, introns, and intergenic regions. As shown in Supplementary Figure S2A, the percentage of sequencing for the exon region was the highest. The distribution trend of m6A methylation sites in the genomes of the three tissues after puberty was similar (Supplementary Figure S2B), and, interestingly, the distribution of ovaries and pituitary glands before puberty was more consistent (Figure 1B). Motif analysis of m6A-modified sequences in the three tissues conformed to the “RRACH” consensus sequence (Figure 1C). These methylation sites were predominantly enriched in termination 3′UTRs and codons (Figure 1D). Interestingly, after puberty, the overall methylation of the pituitary gland was significantly reduced (*p* < 0.01), with no significant changes in other tissues (Figure 1E). However, 922 and 941 differentially methylated genes were detected in the hypothalamus and ovary (Supplementary Tables S4–S6). This may be due to the other two tissues’ genes having equal rates of methylation and demethylation.

### 2.2. Differential Genes of the HPO Axis Before and After Puberty

**Figure 2:**
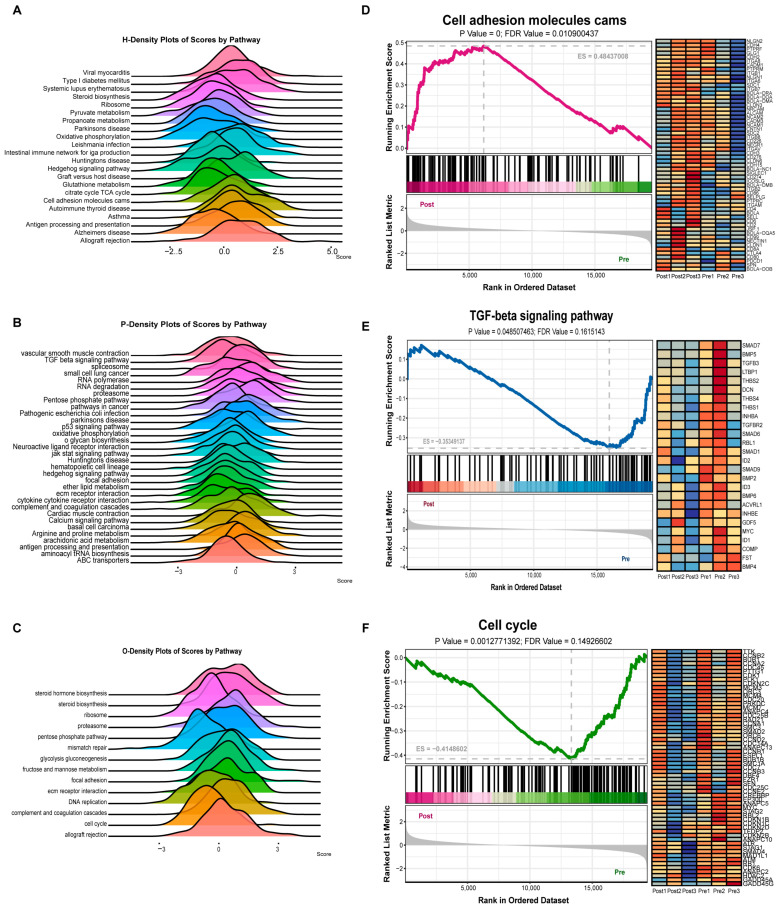
Differential genes of the HPO axis before and after puberty (**A**–**C**) Ridge plot showing the pathways enriched by GSEA analysis between the pre- and post-pubertal groups in bovine hypothalamus (H), pituitary (P), and ovarian (O) samples, with the area of the ridge being indicative of ES. (**D**) Homologous recombination enrichment map (**left**) and heat map of the core gene expression of the pathway (**right**) between the pre- and post-pubertal groups of the hypothalamus. (**E**) Homologous recombination enrichment map (**left**) and heat map of the core gene expression of the pathway (**right**) between the pre- and post-pubertal groups of the pituitary. (**F**) Homologous recombination enrichment map (**left**) and heat map of the core gene expression of the pathway (**right**) between the pre- and post-pubertal groups of the ovary.

The volcano plots show significant differences between the two cohorts, with the hypothalamus, pituitary, and ovaries yielding 176, 261, and 288 differentially expressed genes, respectively (Supplementary Figure S3A). In the GSEA results, ES (enrichment score) indicates the gene enrichment score, NES (normalized enrichment score) reflects the normalized enrichment score, and FDR (false discovery rate) represents the *p*-value adjusted for multiple hypothesis testing. The results were filtered using the criteria of *p* < 0.05 and FDR < 0.25. The ridge plot illustrates the enrichment pathways identified across the three tissues (Figure 2A–C). The cell adhesion molecules (CAMs) pathway associated with neuronal tissue development was significantly activated in the hypothalamus compared to the pre-pubertal period, with BCD6, BOLA-DQA5, CD274, JSP.1, and other genes as its core factors (Figure 2D). The TGF-beta signaling pathway and cell cycle related to cell proliferation were significantly inhibited in the pituitary and ovary, respectively, with BMP4, BMP5, CCNA1, and CDK6 as the core genes, respectively (Figure 2E,F). This may be because the number of cells decreases as the corpus luteum degenerates after puberty. Steroid biosynthesis and steroid hormone synthesis were significantly activated in the ovaries (Supplementary Figure S3B,C).

### 2.3. Differentially Methylated Genes on the HPO Axis Before and After Puberty

**Figure 3:**
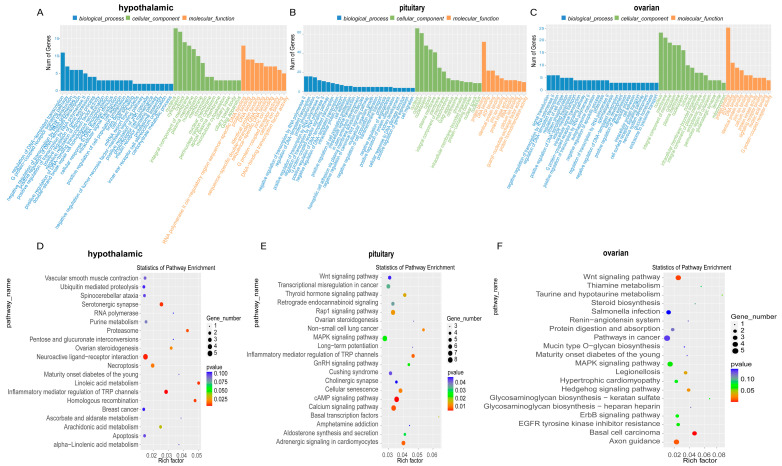
Differentially methylated genes on the HPO axis before and after puberty. (**A**–**C**) GO analysis of the enrichment of differential methylation genes in the hypothalamus, pituitary, and ovarian tissues before and after puberty in biological processes, molecular functions, and cellular components. (**D**–**F**) KEGG analysis of the pathways of significant enrichment of differential methylation genes in the hypothalamus, pituitary, and ovary before and after puberty.

The methylation level of m6A before and after puberty was analyzed (Supplementary Tables S4–S6), and the gene function enrichment was assessed. The results of GO analysis showed that these three tissues were enriched in biological processes, molecular functions, and cellular components, including pathways such as DNA and RNA assembly and translation (Figure 3A–C). The results of KEGG analysis showed that the differential methylation genes in the hypothalamus were enriched in the ovarian steroidogenesis and neuroactive ligand-receptor interaction pathways (Figure 3D), and, in the pituitary, the genes were also enriched in the ovarian steroidogenesis, as well as the Wnt, MAPK, and GnRH signaling, pathways (Figure 3E). In the ovary, the genes were also enriched in the Wnt, MAPK, and steroid biosynthesis pathways (Figure 3F).

### 2.4. Association Analysis of Different m6A Peaks with Differentially Expressed Genes

**Figure 4:**
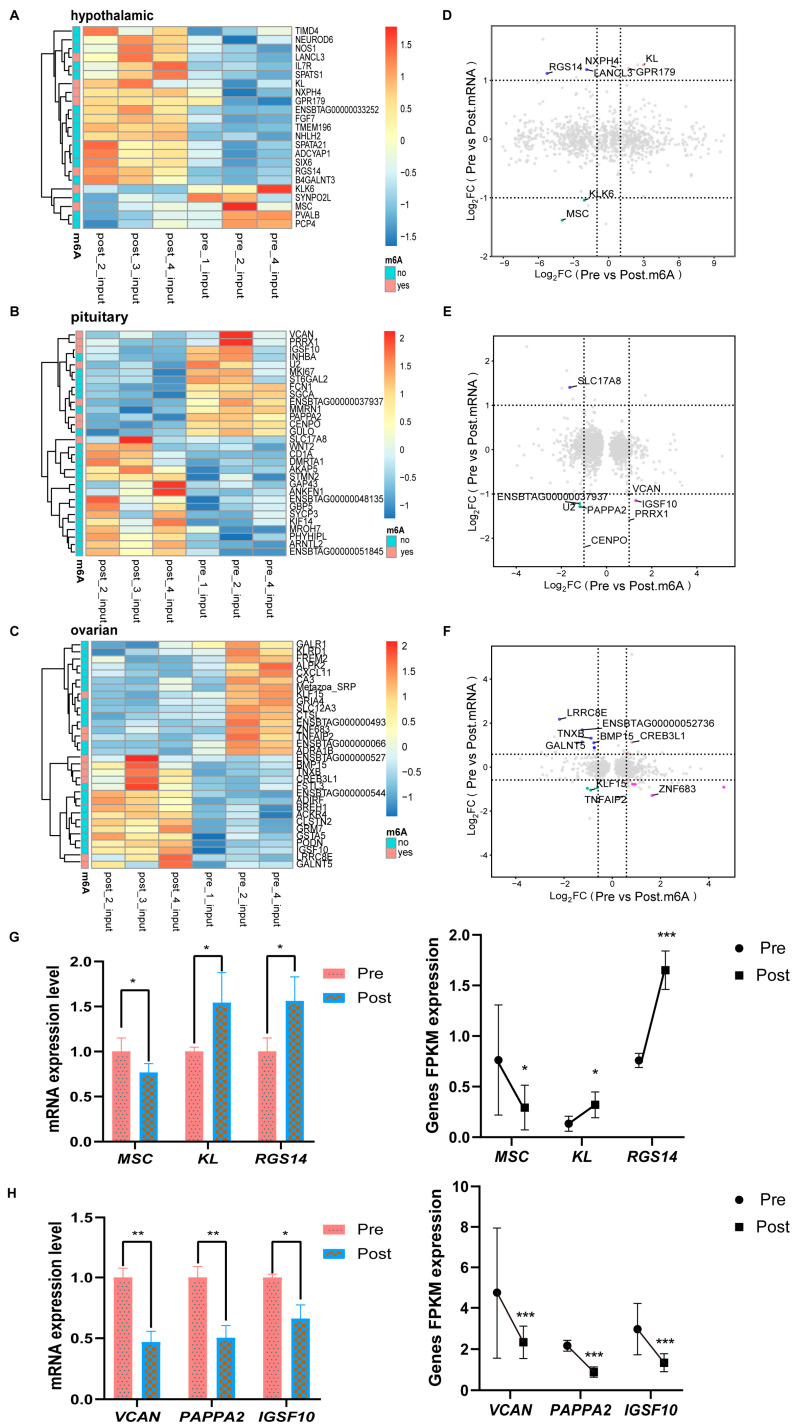
Association analysis of different m6A peaks with differentially expressed genes. (**A**–**C**) Heat maps depicting the methylation expression of differential mRNA in the hypothalamus, pituitary, and ovary before and after puberty. (**D**–**F**) Four-quadrant diagram of shunted expression genes and m6A methylation genes in the hypothalamus, pituitary, and ovary before and after puberty; the abscissa is the logFC value of m6A, and the ordinate is the logFC value of mRNA, with logFC > 1 as the standard. (**G**,**H**) RT-qPCR results of MSC, KL, and RGS14 genes in the hypothalamus and VCAN, PAPPA2, and IGSF10 genes in the pituitary before and after puberty. * *p* < 0.05; ** *p* < 0.01; *** *p* < 0.001.

To further link m6A modification to gene expression, we combined MeRIP-seq and RNA-seq analysis to investigate whether the degree of m6A methylation was associated with mRNA of differentially expressed genes (DEGs). The heat maps show some differentially expressed genes and differentially methylated genes (Figure 4A–C), and the four quadrant diagrams are an intuitive representation of the relationship between gene expression and m6A methylation (Figure 4D–F). The results showed that in the hypothalamus, seven genes were identified as significantly co-differentially expressed, including four hypermethylation genes (mRNA downregulation of MSC, and mRNA upregulation of NXPH4, KL, and GPR179) and three hypomethylation genes (mRNA downregulation of KLK6, and mRNA upregulation of RGS14 and LANCL3). In the pituitary, eight genes were identified as significantly co-differentially expressed, including three hypermethylation genes (mRNA downregulation of IGSF10, VCAN, and PRRX1) and five hypomethylation genes (mRNA upregulation of SLC17A8 and mRNA downregulation of CENPO, PAPPA2, ENSBTAG00000037937, and U2). In the ovary, nine genes were identified as significantly co-differentially expressed, including three hypermethylation genes (ZNF683 and TNFAIP2 mRNA downregulation, and CREB3L1 mRNA upregulation) and six hypomethylation genes (GALNT5, LRRC8E, ENSBTAG00000052736, TNXB, and BMP15 mRNA upregulation, and KLF15 mRNA downregulation). Selected significantly co-differentially expressed genes from the hypothalamus (Figure 4E) and pituitary (Figure 4F) were validated by RT-qPCR, and the results were compared with the transcriptome expression. The expression trend of genes at the RT-qPCR level and the transcriptome level was consistent, further verifying the transcriptome results (Figure 4G,H). 

### 2.5. The Methylation Gene BMP15 Was Differentially Expressed in Ovarian Tissues Before and After Puberty

**Figure 5:**
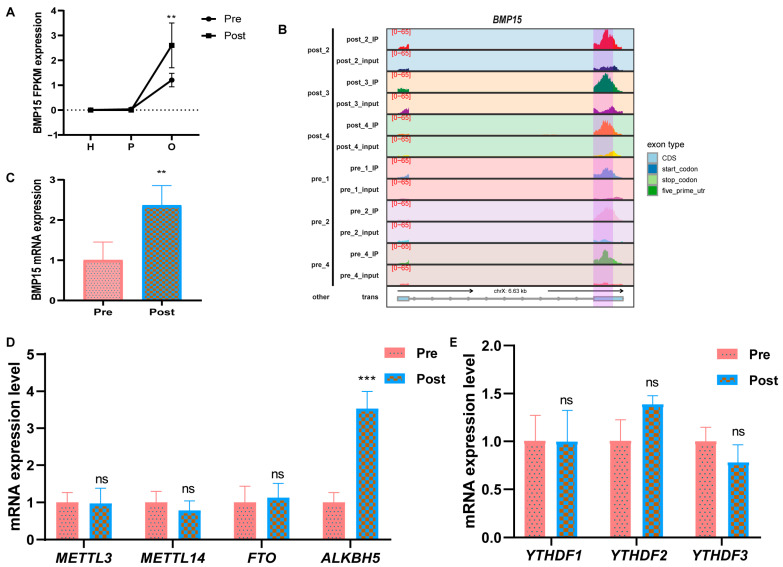
The methylation gene BMP15 was differentially expressed in ovarian tissues before and after puberty. (**A**) The relative gene expression of BMP15 in the hypothalamus (H), pituitary (P), and ovarian (O) before and after puberty. (**B**) The distribution of m6A peaks across the BMP15 transcriptome. (**C**–**E**) The RT-qPCR results of the BMP15, METTL3, MTEEL14, FTO, ALKBH5, and YTHDF1-3 genes in the ovaries before and after puberty. ** *p* < 0.01; *** *p* < 0.001; ns *p* > 0.05.

In the significantly co-differentially expressed genes in the ovary, it has been reported that the gene expression of BMP15 is closely related to follicular development and steroid hormone synthesis, which corresponds to the enrichment analysis pathways described in Section 2.2 and Section 2.3. The transcriptional expression levels of BMP15 in the three tissues were analyzed. It was found that BMP15 was only highly expressed in the ovary (Figure 5A), and the distribution of the m6A peak of BMP15 in the ovary was analyzed (Figure 5B and Supplementary Table S7). The results showed that the m6A modification of BMP15 was downregulated after puberty. The RT-qPCR validation showed that the mRNA expression levels of BMP15 were significantly upregulated (Figure 5C), and the mRNA expression levels of demethylase ALKBH5 were upregulated considerably after the puberty period (Figure 5D). Although there was no significant difference in mRNA expression of the reading protein, there was an upregulated trend of YTHDF2 (Figure 5E), leading us to speculate that the m6A modification of BMP15 may be downregulated by ALKBH5 and recognized by YTHDF2.

### 2.6. The Deficiency of BMP15 Inhibits Ovarian Granulosa Cell Proliferation and Hormone Synthesis in Bovine Ovaries

**Figure 6:**
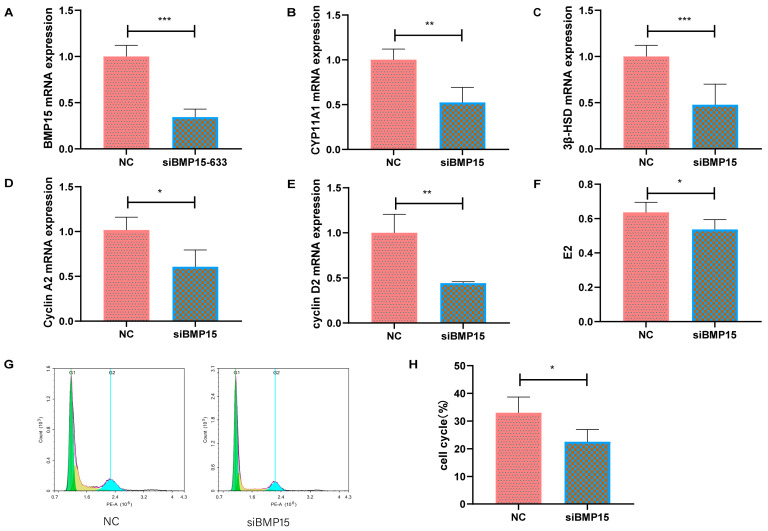
The deficiency of BMP15 inhibits ovarian granulosa cell proliferation and hormone synthesis in bovine ovaries. (**A**–**E**) RT-qPCR results revealed the relative expression of genes in the NC and siBMP15 groups. (**F**) ELISA results unveiled the intracellular levels of estrogen after the inhibition of BMP15. (**G**) Flow cytometry was utilized for assessing the cell cycle. (**H**) The bar chart displays the ratio of GCs in S phases. * *p* < 0.05; ** *p* < 0.01; *** *p* < 0.001.

To further understand the regulatory effect of BMP15 on ovarian cell proliferation and steroid hormone synthesis, we extracted bovine primary ovarian granulosa cells. Following interference with BMP15, mRNA expression levels of BMP15 were examined on bovine ovarian granulosa cells. Compared to the NC group, a notable reduction in the mRNA expression level of BMP15 was evident (Figure 6A). CYP11A1 and 3β-HSD, cyclin A2, and cyclin D2 are key genes for steroid synthesis and cell proliferation, respectively. The mRNA expression levels of CYP11A1, 3β-HSD, cyclin A2, and cyclin D2 were significantly reduced after interference with BMP15 (Figure 6B–E). ELISA results showed that estrogen levels secreted by ovarian granulosa cells were significantly reduced after interfering with BMP15 (Figure 6F). The flow cytometry analysis revealed a significant reduction in the proportion of S-phase cells after interference with BMP15 (Figure 6G,H). These results indicated that the reduction of BMP15 inhibited the proliferation and hormone secretion of bovine ovarian granulosa cells.

### 2.7. BMP15-Dependent m6A Modification of ALKBH5 Regulates Granulosa Cell Proliferation and Hormone Secretion

**Figure 7:**
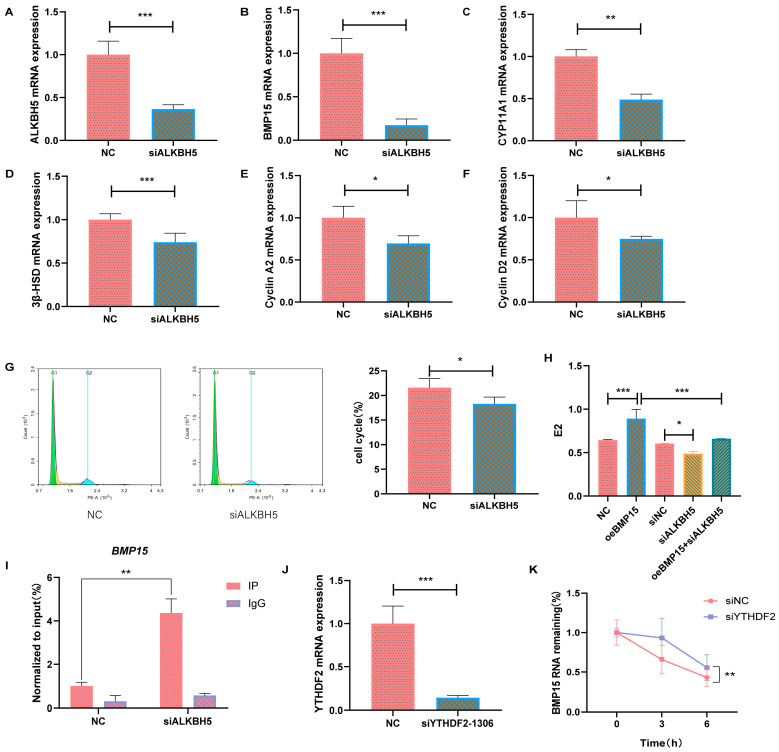
BMP15-dependent m6A modification of ALKBH5 regulates granulosa cell proliferation and hormone secretion (**A**–**F**) RT-qPCR results revealed the relative expression of genes in the NC and siALKBH5 groups. (**G**) Flow cytometry was utilized for assessing the cell cycle and the bar chart displays the ratio of GCs in S-phases. (**H**) ELISA results unveiled the intracellular levels of estrogen after the overexpression of BMP15 or interference with ALKBH5. (**I**) Determination of m6A modification level of BMP15 mRNA after inhibition of ALKBH5 using MeRIP-RT-qPCR. (**J**) RT-qPCR results revealed the relative expression of YTHDF2 in the NC and siYTHDF2 groups. (**K**) The degradation rate of BMP15 mRNA was determined via RT-qPCR analysis at specified time points after treatment with actinomycin D in granulosa cells following YTHDF2 inhibition. * *p* < 0.05; ** *p* < 0.01; *** *p* < 0.001.

To clarify the effect of m6A modification of BMP15 on bovine ovarian granulosa cells, ALKBH5 was interfered with. In contrast to the NC group, the mRNA expression levels of ALKBH5 and BMP15 in the siALKBH5 group exhibited substantial reductions (Figure 7A,B), accompanied by significantly diminished mRNA expression levels of CYP11A1, 3β-HSD, cyclin A2, and cyclin D2 (Figure 7C–F). Flow cytometry analysis demonstrated a notable reduction in the proportion of S-phase cells after the interference with ALKBH5 (Figure 7G). The ELISA results revealed markedly elevated estrogen levels in the oeBMP15 group, and, conversely, significantly reduced estrogen levels in the siALKBH5 group, in comparison to the control group. Furthermore, the oeBMP15 + siALKBH5 group exhibited markedly reduced estrogen levels in contrast to the oeBMP15 group (Figure 7H). The MeRIP-RT-qPCR results indicated a significant increase in the m6A modification of BMP15 subsequent to the interference with ALKBH5, thus suggesting BMP15 as a potential target for ALKBH5 (Figure 7I). BMP15 gene level and methylation level were upregulated and downregulated, respectively. Given that YTHDF2 promotes mRNA degradation by selectively binding to m6A modified mRNA, we speculate that it may recognize and degrade BMP15 that undergoes m6A modification. To validate our hypothesis, YTHDF2 was first interfered with; the mRNA level of YTHDF2 was significantly downregulated (Figure 7J). Then we assessed the stability of BMP15 mRNA following interference with YTHDF2. Remarkably, the results demonstrated that the downregulation of YTHDF2 led to a delayed decay of BMP15 m6A-methylated transcripts (Figure 7K). 

## 3. Discussion

Puberty is a critical biological process that profoundly influences reproductive and developmental outcomes in mammals. Early or late onset of puberty can significantly impact the economic performance of cattle farming [15]. The HPO axis plays a pivotal role in driving puberty [9]. Recent evidence suggests that N6-methyladenosine (m6A) modifications may play a crucial role in regulating hormone secretion along the HPO axis [16,17,18]. Despite this, m6A profiles within the HPO axis during the onset of puberty have not been extensively characterized. Previous studies have shown that m6A peaks are primarily concentrated on stop codons, long exons, and 3′ untranslated regions (UTRs) [19,20]. Our results confirm that these peaks are predominantly located in the exon region. The alignment of these peaks with introns and intergenic regions might be attributed to pre-mRNA, incomplete genome annotation, splicing events, or background noise. The m6A motif “RRACH” is overexpressed in the m6 A motif region [21], a finding corroborated by our data. Identification of these motifs is critical to understanding the regulatory mechanisms of gene expression and also provides insights into the significant presence of m6A methylation in the bovine HPO axis at the onset of puberty.

Gene Set Enrichment Analysis (GSEA) of hypothalamic samples before and after puberty reveals the activation of cell adhesion molecules (CAMs). CAMs are known to participate in cell-to-cell communication [22], regulate the hypothalamic–pituitary–adrenal (HPA) axis response to immune stimulation [23], and influence human growth hormone secretion [24]. Studies have shown a negative correlation between CAMs and estradiol regulation [25], which aligns with our findings of decreased estradiol levels and upregulated CAMs after puberty. The interaction between the TGF-β signaling pathway and follicle-stimulating hormone (FSH) secreted by the pituitary promotes the growth of granulosa cells in vitro and protects the follicles from atresia in vivo [26,27]. Our results show that the cell cycle is suppressed after puberty in the ovaries, which may regulate progesterone secretion by the corpus luteum [28]. Additionally, the differential methylation genes in the hypothalamus and pituitary before and after puberty are enriched in the ovarian steroid hormone signaling pathway, while those in the pituitary and ovaries are enriched in the MAPK signaling pathway. The MAPK signaling pathway can mediate estrogen receptor activation and promote cell proliferation [29,30,31,32], underscoring the importance of m6A modification in the initiation of puberty.

Our sequencing results identified several differentially significant genes in the hypothalamus and pituitary before and after puberty, including RGS14, LANCL3, MSCNXPH4, KL, GPR179, SLC17A8, IGSF10, VCAN, PRRX1, CENPO, PAPPA2, ENSBTAG00000037937, and U2. For instance, KL regulates growth hormone (GH) secretion from the pituitary gland [33] and is associated with growth factor 1 (IGF-1) and rapid-onset central precocious puberty (RP-CPP), which may be relevant for female precocious puberty [34]. IGSF10 is a marker gene for delayed puberty [35], and mutations in genes like IGSF10 and FTO have been identified in patients with congenital gonadotropin hypogonadism [36]. PAPPA2 modulates the binding of insulin-like growth factor-binding proteins (IGFBPs) to IGF-1, thereby promoting IGF-1 bioavailability [37]. Females with higher IGF-1 expression tend to experience earlier menarche, while those with IGF-1 mutations experience delayed puberty [38]. Our results indicate that mRNA and m6A levels of KL in the hypothalamus are significantly upregulated after puberty, the mRNA and m6A levels of IGSF10 in pituitary tissue are significantly downregulated after puberty, and mRNA levels of PAPPA2 decrease but m6A levels are upregulated after puberty. The reciprocal interaction between KL and PAPPA2 with IGF-1 suggests their involvement in the negative feedback regulation of the pituitary to the hypothalamus, although the specific mechanisms require further exploration.

Among the ovarian differential methylation genes before and after the first puberty, BMP15, a member of the TGF-β family, participates in the regulation of cell proliferation [39,40,41], steroid hormone synthesis [42,43], and the MAPK signaling pathway [44]. Our study demonstrates that the m6A modification of BMP15 is significantly downregulated in post-pubertal ovaries, potentially due to the upregulation of the demethylase ALKBH5. Interfering with BMP15 inhibits bovine ovarian granulosa cell proliferation and blocks steroid synthesis in granulosa cells, resulting in a decrease in estradiol production. ALKBH5 is known to modulate mRNA export and RNA metabolism by reducing m6A levels in nuclear speckles [45], and plays a crucial role in spermatogenesis [46]. Our results show that interfering with ALKBH5 significantly reduces the proliferation, estrogen expression, and steroid synthesis in ovarian granulosa cells consistent with the effects of BMP15 interference; MeRIP-RT-qPCR analysis further supports the dependence of BMP15 m6A modification on ALKBH5.

The YTH family of m6A reader proteins, including YTHDF1-3, YTHDC1, and YTHDC2, plays a significant role in mRNA stability and translation efficiency. YTHDF2, in particular, facilitates mRNA degradation by selectively recognizing m6A-containing transcripts [47]. In our study, we found that the mRNA level of BMP15 was significantly upregulated but its m6A modification level was significantly downregulated in ovaries before and after puberty. We hypothesized that the upregulation of BMP15 mRNA might be due to a reduction in its m6A modification level, thereby decreasing its recognition and degradation by YTHDF2. Indeed, interfering with YTHDF2 slowed down the RNA decay of BMP15, supporting our hypothesis. Further investigation using RNA immunoprecipitation (RIP) and other experiments is warranted to elucidate the interaction between YTHDF2 and BMP15. 

## 4. Materials and Methods

### 4.1. Animal Selection

Crossbred offspring were obtained from Dehongyingrui Animal Husbandry Co., Ltd. (Dehong Dai, China), with zebu cattle as the mothers and red Angus cattle as the fathers. Pubertal status was determined by ultrasonography and ultrasound screening for the first corpus luteum (CL). Puberty was monitored every two weeks. Three heifers were euthanized during the luteal phase of their second estrus cycle post-puberty. When the first heifer in a group of 6 young heifers reached first puberty, 3 pre-pubertal young heifers were randomly selected from the remaining heifers that had never ovulated to obtain tissue samples. Progesterone levels in the heifer serum were detected using an ELISA kit (MEIMIAN, Wuhan, China) prior to slaughter (Supplementary Figure S1). The weight and age of each heifer at slaughter were recorded (Supplementary Table S8).

### 4.2. Hypothalamus, Pituitary, and Ovarian Tissue Samples

Following ethical guidelines, the hypothalamus, the entire pituitary gland, and the ovaries were removed immediately after slaughter. Immediately following decapitation, the hypothalamic region was dissected, the cerebellum was removed, and the junction between the brainstem and the brain was exposed. The hypothalamus was then cut along the root, and the entire pituitary gland was removed from the pituitary fossa. The ovaries were also removed. All tissues were flash-frozen in liquid nitrogen and stored at −80 °C pending subsequent sequencing.

### 4.3. Extraction of Total RNA

Total RNA from the samples were using TRIzol (Invitrogen, Carlsbad, CA, USA). The quantity of the total RNA was assessed using a NanoDrop ND-1000 spectrophotometer (NanoDrop, Wilmington, DE, USA), and integrity analysis was conducted using the Bioanalyzer 2100 system (Agilent, Santa Clara, CA, USA). RNA samples were considered suitable for downstream procedures if they met the following criteria: RNA concentration > 50 ng/μL, RNA Integrity Number (RIN) > 7.0, OD260/280 ratios > 1.8, and total RNA quantity > 50 μg. The RNA was fragmented using oligonucleotide (dT) magnetic beads (Dynabeads Oligo (dT), Cat. No. 25-61005, Thermo Fisher, Waltham, MA, USA), followed by specific capture using the NEBNext^®^ Magnesium RNA Fragmentation Module (Cat. No. E6150S, USA) at 94 °C (mRNA-seq) or 86 °C (MeRIP-seq) for 7 min.

### 4.4. mRNA-Seq and MeRIP-Seq

RNA fragments were reverse transcribed using SuperScript™ II Reverse Transcriptase (Invitrogen, cat. 1896649, USA) to produce cDNA. Second-stranded DNAs labeled with uracil (U) were synthesized using E. coli DNA polymerase I (NEB, cat.m0209, Ipswich, MA, USA), RNase H (NEB, cat.m0297, USA), and dUTP Solution (Thermo Fisher, cat. R0133, USA). Terminal A-bases were added to the ends of the strands to enable ligation, which featured a T-base overhang. Adapters were either single- or dual-indexed, followed by size selection using AMPureXP beads. Post-ligation, both processes involved treatment with a heat-labile UDG enzyme (NEB, cat.m0280, USA) before PCR amplification. PCR conditions included an initial denaturation step at 95 °C for 3 min, followed by 8 cycles of denaturation at 98 °C for 15 s, annealing at 60 °C for 15 s, and extension at 72 °C for 30 s, with a final extension step at 72 °C for 5 min. The resulting cDNA libraries had an average insert size of 300 ± 50 bp. Paired-end sequencing (PE150) was conducted on an Illumina Novaseq™ 6000 platform (LC-Bio Technology CO., Ltd., Hangzhou, China) following the vendor’s recommended protocols.

### 4.5. mRNA-Seq and MeRIP-Seq Data Analysis

Fastp software (Version: 0.19.4; accessed on 6 March 2021; https://github.com/OpenGene/fastp) was used to remove the reads that contained adaptor contamination, low quality bases, and undetermined bases with default parameters. The sequence quality of IP and Input samples were verified using FastQC (Version: 1.5.0; accessed on 8 April 2021; https://www.bioinformatics.babraham.ac.uk/projects/fastqc/) and RseQC (Version: 1.5.0; accessed on 1 May 2021; http://rseqc.sourceforge.net/). Then, we used HISAT2 (Version: 2.0.4; accessed on 27 May 2021; http://daehwankimlab.github.io/hisat2) to map reads to the reference genome bos taurus (Version: v107). Peak calling and diff peak analysis were performed using the R package exomePeak (Version: 1.5.0; accessed on 6 June 2021; https://www.bioconductor.org/packages/3.3/bioc/html/exomePeak.html), and peaks were annotated by intersection with gene architecture using the R package ANNOVAR (accessed on 27 June 2021; http://www.openbioinformatics.org/annovar/). HOMER (Version: 4.10; accessed on 29 July 2021; http://homer.ucsd.edu/homer/motif) was used for de novo and known motif finding followed by localization of the motif with respect to peak summit. StringTie (Version: 2.1.2; accessed on 16 August 2021; https://ccb.jhu.edu/software/stringtie) was used to determine expression level for all transcripts and genes from input libraries by calculating FPKM (total exon fragments/mapped reads (millions) × exon length (kB)). The differentially expressed transcripts and genes were selected with log2 (fold change) ≥ 1 or log2 (fold change) ≤ −1 and *p* value < 0.05 using the R package edgeR (Version: 4.1; accessed on 10 October 2021; https://bioconductor.org/packages/edgeR). Pathway enrichment on all screened genes was performed using OmicStudio (accessed on 10 August 2022; www.omicstudio.cn).

### 4.6. Primary Cell Culture

Ovaries from healthy bovines were immersed in normal saline at 37 °C and promptly transported to the laboratory within a four-hour window. Upon arrival, the ovaries underwent six cycles of washing with normal saline containing penicillin/streptomycin (Sangon Biotech, Shanghai, China) before subsequent extraction within the cell culture chamber. In the cell culture chamber, follicular fluid was aspirated from the 3–8 mm follicles using a 5 mL syringe. The aspirated follicular fluid was centrifuged in a 5 mL tube at 1000 rpm for 5 min. Cells were washed with PBS containing penicillin/streptomycin. The collected granulosa cells were cultured in 5% CO_2_, 37 °C in DMEM/F-12 medium (Gibco, Waltham, MA, USA).

### 4.7. Transient Cell Transfection

Transfection was initiated once the granulosa cells reached 70% confluence. The siRNAs (Supplementary Table S9) and oeRNA were manufactured by JTS Scientific (Wuhan, China). Specifically, the NC and siRNAs were centrifuged at 3000 rpm for 3 min, dissolved in a DEPC-treated solution, and gently mixed. Then, 10–15 μL of the dissolved NC, siRNA, and 5 μL of Lipofectamine™ 2000 (Thermo Fisher Scientific, Waltham, MA, USA) were added to 500 μL of DMEM/F12 medium in a 1.5 mL centrifuge tube. After incubating for 5 min, the Lipofectamine™ 2000 complexes were added to the NC or siRNA complex, mixed thoroughly, and left for 20 min. The culture medium was removed from the cell culture dish and cells were washed three times with PBS buffer. Then, 4 mL of DMEM/F12 medium along with the prepared complex were added to each dish. After 6 h of incubation at 37 °C, 550 μL of fetal bovine serum was added to each dish. Overexpression of adenovirus was achieved by adding the appropriate multiplicity of infection (MOI) of virus to the cell culture medium, replacing the culture medium after 12 h of infection, and waiting for the virus to infiltrate the cells for 24–48 h for subsequent processing.

### 4.8. Realtime Fluorescence Quantitative PCR (RT-qPCR)

Total RNA was extracted from cellular pellets using Trizol reagent (Takara, Tokyo, Japan), followed by reverse transcription to cDNA employing the PrimeScript™ RT kit (Takara, Tokyo, Japan). For real-time fluorescence quantification, SYBR^®^ Premix Ex Taq™ II (Takara, Tokyo, Japan) was employed. Analysis of relative gene expression was carried out utilizing the 2^−ΔΔCt^ comparison method, with primer sequences detailed in Supplementary Table S10. 

### 4.9. MeRIP-RT-qPCR

All programs were used following the manufacturers’ guidelines, including immune capture lysis, enriched RNA release and recovery, IP-m6A reverse transcription, qPCR detection, etc. The MeRIP ™ M6A assay kit (A-P-9018 class, IVDSHOW, Zhangjiakou, China) was used. The primer sequences are detailed in Supplementary Table S11.

### 4.10. Cell Cycle Assay

Cells were pelleted and fixed in 75% ethanol, centrifuged after incubation at 4 °C for 2 h, and PBS was added to resuspend the washed cells. The cleaned granulosa cells were incubated with PI (BD Biosciences, San Jose, CA, USA) at 37 °C for 30 min, and then the cell cycle was measured by flow cytometry (ACEA Biosciences, Hangzhou, China).

### 4.11. ELISA

After disrupting the cells, the cells were repeatedly frozen and thawed to obtain an intracellular suspension. The suspension was centrifuged at 4 °C, 3000 rpm for 15 min, and the supernatant was carefully collected. Intracellular components were analyzed using an ELISA kit (MEIMIAN, Jiangsu, China) following the manufacturer’s instructions.

### 4.12. mRNA Stability Assays

Interfering cells were incubated with actinomycin D (SBR00013, Sigma, Kawasaki, Japan) for 0 h, 3 h, or 6 h, followed by RNA extraction. The half-life of BMP15 mRNA was analyzed by quantitative RT-qPCR as previously described.

### 4.13. Statistical Analysis

Statistical analysis was conducted using GraphPad Prism 8 (GraphPad Software, Inc., San Diego, CA, USA). Data are presented as mean ± standard deviation. For comparisons involving more than two groups, one-way analysis of variance (ANOVA) and Tukey’s post hoc test were employed, while the Student’s *t*-test was utilized for comparisons between two groups. Statistical significance was defined as *p* < 0.05. Each experiment was independently repeated three times.

## 5. Conclusions

This study analyzes N6-methyladenosine (m6A) modifications in the hypothalamus, pituitary, and ovaries during bovine puberty. Our findings suggest that signaling pathways such as cell adhesion molecules (CAMs), TGF-β, cell cycle, steroid synthesis, and MAPK, as well as m6A modifications of genes such as KL, IGSF10, PAPPA2, and BMP15, may modulate the function of the hypothalamus, pituitary, and ovarian at the onset of puberty in bovines. Specifically, the m6A modification of BMP15 appears to depend on the demethylase ALKBH5, which recognizes and modifies BMP15 through the reader protein YTHDF2, leading to a downregulation of its methylation level in the ovaries after puberty. Moreover, the methylation modification of BMP15 can promote estrogen secretion, steroid production, and cell proliferation in ovarian granulosa cells. These findings represent significant advances in understanding the regulatory role of epigenetic modifications, such as m6A, in the onset of puberty in bovine.

## Data Availability

The data were uploaded to NCBI: GSE277594, GSE277975, GSE279563.

## Supplementary Materials

The following supporting information can be downloaded at: https://www.mdpi.com/article/10.3390/ijms252111605/s1.
