# Supplementary material for: ALKBH5 Reduces BMP15 mRNA Stability and Regulates Bovine Puberty Initiation Through an m6A-Dependent Pathway
Source: Int J Mol Sci. 2024 Oct 29;25(21):11605. doi: 10.3390/ijms252111605 (PMC11546126; doi:10.3390/ijms252111605)
Supplement: Supplementary file 1 [file ijms-25-11605-s001.zip › Supplementary Figure.pdf]

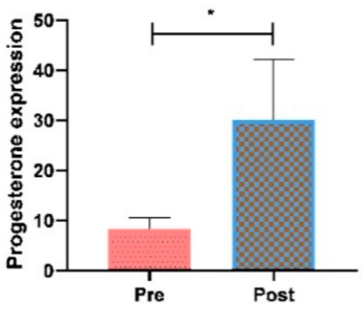

Supplementary Figure S1 Progesterone levels in serum

The ELISA results revealed the levels of progesterone in calves around puberty.

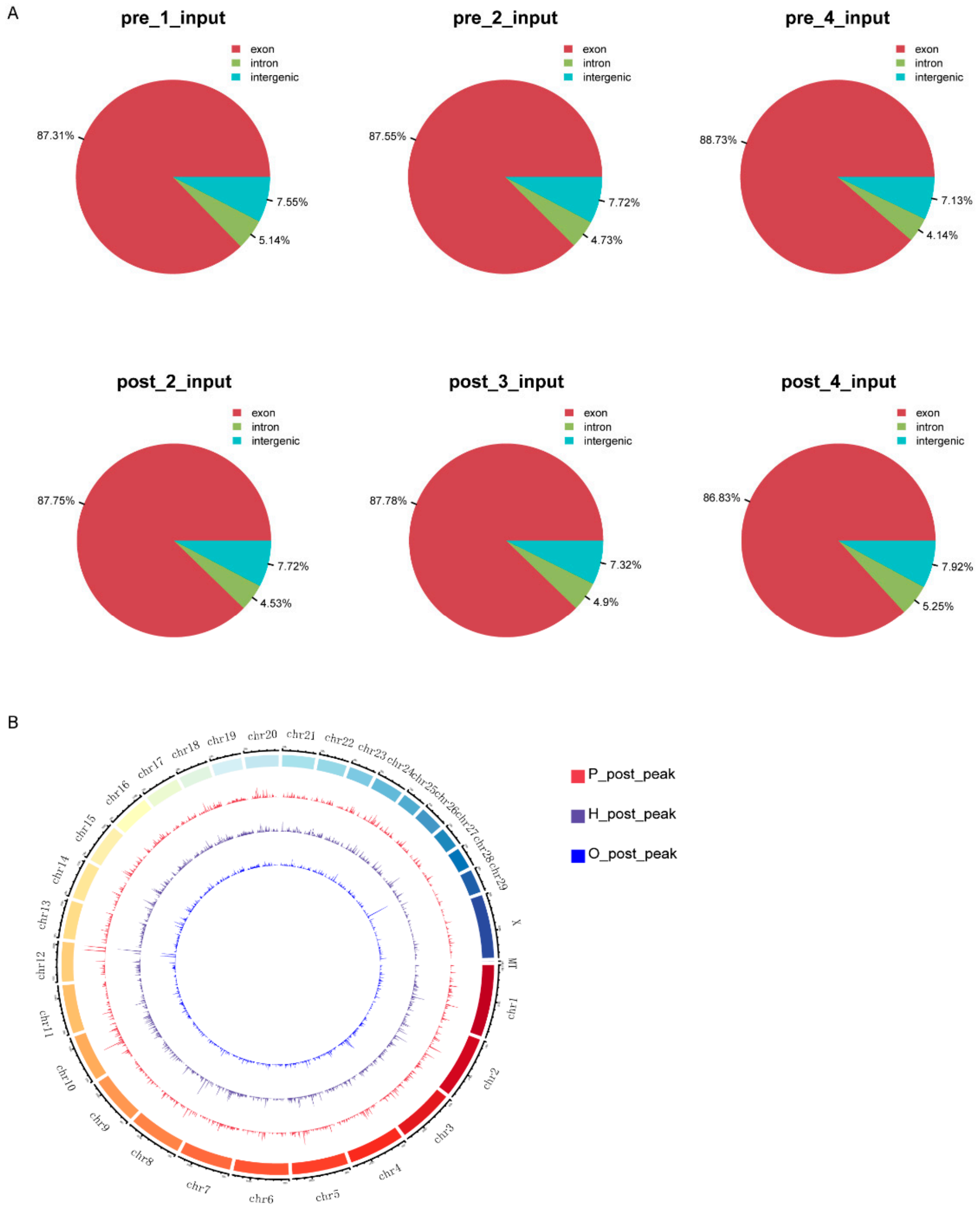

Supplement Figure S2. HPO axis-sequencing quality control and identification of peaks before and after puberty

(A) The valid data for each set were matched to a reference genome defined as exon, intron, and intergene matching.

(B) The distribution of m6A peak sites on the bovine genome was obtained from samples from three tissues after the first puberty. The outermost circle represents the distribution of chromosomes on the genome, the red circle represents the pituitary gland, the purple circle represents the hypothalamus, and the blue circle represents the ovary.

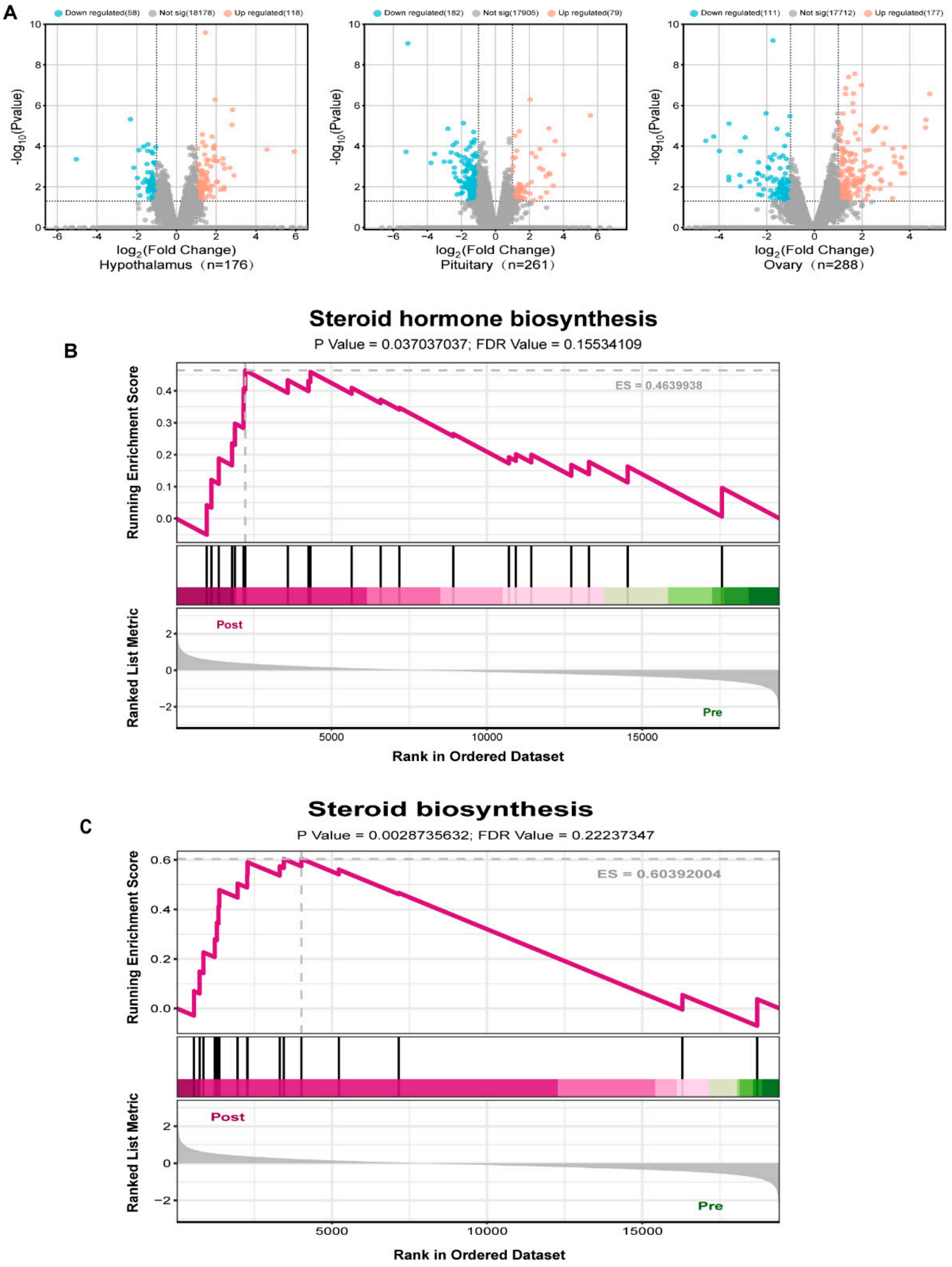

Supplement Figure S3. The differential genes of the HPO axis before and after puberty were analyzed

(A) volcano map illustrating the differential expression of genes between the pre- and post-groups in bovine hypothalamus, pituitary, and ovarian samples.

(B-C) The homologous recombination enrichment map between the pre-and post-groups of the ovary.
