# Supplementary material for: ALKBH5 Reduces BMP15 mRNA Stability and Regulates Bovine Puberty Initiation Through an m6A-Dependent Pathway
Source: Int J Mol Sci. 2024 Oct 29;25(21):11605. doi: 10.3390/ijms252111605 (PMC11546126; doi:10.3390/ijms252111605)
Supplement: Supplementary file 1 [file ijms-25-11605-s001.zip › Supplementary Table.pdf]

Supplementary Table S1. The comparison of hypothalamic reference genome reads statistics

Sample	Valid reads	Mapped reads	Unique Mapped reads	Multi Mapped reads
pre_1_IP	49453450	48139400(97.34%)	37239279(75.30%)	10900121(22.04%)
pre_2_IP	51686978	50290026(97.30%)	38545363(74.57%)	11744663(22.72%)
pre_4_IP	47336558	46022304(97.22%)	34668864(73.24%)	11353440(23.98%)
post_2_IP	52174828	50502253(96.79%)	38668403(74.11%)	11833850(22.68%)
post_3_IP	51901920	50259188(96.83%)	38819945(74.79%)	11439243(22.04%)
post_4_IP	54040754	52356195(96.88%)	40977461(75.83%)	11378734(21.06%)
pre_1_input	50258444	49305998(98.10%)	39457187(78.51%)	9848811(19.60%)
pre_2_input	53358718	52359059(98.13%)	41596530(77.96%)	10762529(20.17%)
pre_4_input	50294580	49348742(98.12%)	38453749(76.46%)	10894993(21.66%)
post_2_input	51437638	50404007(97.99%)	39432277(76.66%)	10971730(21.33%)
post_3_input	51007668	49991664(98.01%)	39509668(77.46%)	10481996(20.55%)
post_4_input	49141658	48137468(97.96%)	38389773(78.12%)	9747695(19.84%)

Supplementary Table S2. The comparison of pituitary reference genome reads statistics

Sample	Valid reads	Mapped reads	Unique Mapped reads	Multi Mapped reads
pre_1_IP	37979692	36922959(97.22%)	29649265(78.07%)	7273694(19.15%)
pre_2_IP	40559966	39368728(97.06%)	31768830(78.33%)	7599898(18.74%)
pre_4_IP	38030336	36971804(97.22%)	28872408(75.92%)	8099396(21.30%)
post_2_IP	38994330	37909742(97.22%)	30057758(77.08%)	7851984(20.14%)
post_3_IP	38556512	37507913(97.28%)	29628132(76.84%)	7879781(20.44%)
post_4_IP	38220372	37146691(97.19%)	29380945(76.87%)	7765746(20.32%)
pre_1_input	41134750	40294767(97.96%)	31686562(77.03%)	8608205(20.93%)
pre_2_input	43543392	42666228(97.99%)	32843426(75.43%)	9822802(22.56%)
pre_4_input	39860816	39039242(97.94%)	30660930(76.92%)	8378312(21.02%)
post_2_input	41144218	40271207(97.88%)	31558595(76.70%)	8712612(21.18%)
post_3_input	38853468	38040322(97.91%)	29898796(76.95%)	8141526(20.95%)
post_4_input	42366108	41550248(98.07%)	32985635(77.86%)	8564613(20.22%)

Supplementary Table S3. The comparison of ovary reference genome reads statistics

Sample	Valid reads	Mapped reads	Unique Mapped reads	Multi Mapped reads
post_2_input	39757310	38953739(97.98%)	31289265(78.70%)	7664474(19.28%)
post_2_IP	40001034	38860709(97.15%)	31731374(79.33%)	7129335(17.82%)
post_3_input	41760506	40927049(98.00%)	32764337(78.46%)	8162712(19.55%)
post_3_IP	37562414	36507372(97.19%)	29652101(78.94%)	6855271(18.25%)
post_4_input	40257492	39387926(97.84%)	31607859(78.51%)	7780067(19.33%)
post_4_IP	38949936	37827138(97.12%)	30810376(79.10%)	7016762(18.01%)
pre_1_IP	38721768	37687071(97.33%)	30209487(78.02%)	7477584(19.31%)
pre_1_input	41513710	40709445(98.06%)	32479930(78.24%)	8229515(19.82%)
pre_2_IP	39453714	38421261(97.38%)	30976394(78.51%)	7444867(18.87%)
pre_2_input	40725904	39899515(97.97%)	31624218(77.65%)	8275297(20.32%)
pre_4_input	41912904	41014461(97.86%)	32871253(78.43%)	8143208(19.43%)
pre_4_IP	39551940	38447409(97.21%)	31346253(79.25%)	7101156(17.95%)

Supplementary Table S4. The hypothalamic differentially methylated genes

sort	geneName	Peak Annotation	geneID	gene_chr
1	ENSBTAG00000034496	exonic	ENSBTAG00000034496	chr22
2	ATP6	exonic	ENSBTAG00000043584	MT
3	SEM1	UTR3	ENSBTAG00000052508	chr4
4	DNAJA1	UTR3	ENSBTAG00000016265	chr8
5	POLR1D	UTR3	ENSBTAG00000008642	chr12
6	COPS2	UTR3	ENSBTAG00000013425	chr10
7	ENSBTAG00000054942	exonic	ENSBTAG00000054942	X
8	PPM1B	UTR5	ENSBTAG00000000223	chr11
9	CAVIN4	UTR5	ENSBTAG00000021992	chr8
10	TMEFF1	UTR3	ENSBTAG00000021991	chr8
11	FBXL19	UTR5	ENSBTAG00000040555	chr25
12	DNAJA1	exonic	ENSBTAG00000016265	chr8
13	ASNSD1	UTR3	ENSBTAG00000000492	chr2
14	HNRNPA3	exonic	ENSBTAG00000021580	chr2
15	TADA2A	UTR3	ENSBTAG00000014677	chr19
16	ENSBTAG00000043974	UTR3	ENSBTAG00000043974	chr25
17	CXXC5	UTR3	ENSBTAG00000003986	chr7
18	RUVBL1	UTR3	ENSBTAG00000020998	chr22
19	ZDHHC7	UTR3	ENSBTAG00000002639	chr18
20	ENSBTAG00000031687	exonic	ENSBTAG00000031687	chr18
21	TTYH2	UTR3	ENSBTAG00000011007	chr19
22	ATG13	UTR3	ENSBTAG00000017325	chr15
23	ENSBTAG00000027426	exonic	ENSBTAG00000027426	chr16
24	FZR1	UTR3	ENSBTAG00000031387	chr7
25	ACTR1A	UTR3	ENSBTAG00000021067	chr26
26	ACSL6	UTR3	ENSBTAG00000019708	chr7
27	CDIP1	UTR3	ENSBTAG00000018938	chr25
28	CLIP1	exonic	ENSBTAG00000016779	chr17
29	ACBD3	UTR3	ENSBTAG00000013128	chr16
30	ZNF239	exonic	ENSBTAG00000019039	chr28
31	SYP	UTR3	ENSBTAG00000016096	X
32	MAP2K1	UTR5	ENSBTAG00000033983	chr10
33	TMEM97	UTR3	ENSBTAG00000008109	chr19
34	COPS2	UTR3	ENSBTAG00000013425	chr10
35	EEF1AKMT1	ncRNA_exonic	ENSBTAG00000008296	chr12
36	UFM1	UTR3	ENSBTAG00000051486	chr12
37	NAP1L4	UTR5	ENSBTAG00000022160	chr29
38	ENSBTAG00000054466	exonic	ENSBTAG00000054466	chr6
39	EIF4B	UTR3	ENSBTAG00000006883	chr5
40	UBOX5	UTR3	ENSBTAG00000011007	chr19

Supplementary Table S5. The pituitary differentially methylated genes

sort	geneName	Peak Annotation	geneID	gene_chr
1	ATP6	exonic	ENSBTAG00000043584	MT
2	GNAS	-	ENSBTAG00000052413	chr13
3	GNAS	UTR3	ENSBTAG00000047223	chr13
4	RPS25	UTR3	ENSBTAG00000027772	chr15
5	RPL4	UTR3	ENSBTAG00000005211	chr10
6	MRFAP1L1	UTR3	ENSBTAG00000046936	chr6
7	UBB	UTR5	ENSBTAG00000017246	chr19
8	RPL7	UTR3	ENSBTAG00000020139	chr14
9	FTH1	UTR5	ENSBTAG00000011184	chr29
10	COL18A1	exonic	ENSBTAG00000023907	chr1
11	NFE2L1	UTR3	ENSBTAG00000013653	chr19
12	ENSBTAG00000034185	UTR3	ENSBTAG00000034185	chr18
13	ALDH3A2	UTR3	ENSBTAG00000039161	chr19
14	CCN3	UTR3	ENSBTAG00000002080	chr14
15	RPS24	UTR3	ENSBTAG00000013264	chr28
16	SEC61A1	UTR3	ENSBTAG00000004937	chr22
17	TNKS1BP1	exonic	ENSBTAG00000016469	chr15
18	HIPK3	UTR3	ENSBTAG00000014884	chr15
19	ANKRD17	exonic	ENSBTAG00000004912	chr6
20	CREG1	UTR3	ENSBTAG00000008931	chr3
21	CHMP3	UTR5	ENSBTAG00000013589	chr11
22	ENSBTAG00000027426	exonic	ENSBTAG00000027426	chr16
23	KIAA0513	UTR3	ENSBTAG00000020835	chr18
24	LZTS2	exonic	ENSBTAG00000003298	chr26
25	JMJD1C	exonic	ENSBTAG00000001573	chr28
26	SLF2	exonic	ENSBTAG00000012077	chr26
27	NCL	UTR3	ENSBTAG00000016174	chr2
28	NRDC	exonic	ENSBTAG00000021174	chr3
29	ATXN7L3B	UTR3	ENSBTAG00000035083	chr5
30	DGCR2	exonic	ENSBTAG00000000429	chr17
31	MICOS10	exonic	ENSBTAG00000050219	chr2
32	UQCRH	UTR5	ENSBTAG00000009603	chr3
33	UBE2D3	UTR3	ENSBTAG00000048761	chr6
34	WDR6	exonic	ENSBTAG00000018910	chr22
35	TCAF1	UTR3	ENSBTAG00000002554	chr4
36	CCDC47	exonic	ENSBTAG00000011834	chr19
37	ALKBH5	exonic	ENSBTAG00000025046	chr19
38	PNRC2	UTR3	ENSBTAG00000030435	chr2
39	CLINT1	exonic	ENSBTAG00000016199	chr7
40	ENSBTAG00000052260	exonic	ENSBTAG00000052260	chr15

Supplementary Table S6. The ovary differentially methylated genes

sort	geneName	Peak Annotation	geneID	gene_chr
1	TCEAL8	UTR3	ENSBTAG00000027843	X
2	SLITRK2	exonic	ENSBTAG00000039377	X
3	RPL15	UTR3	ENSBTAG00000033080	chr27
4	SMIM13	UTR3	ENSBTAG00000046160	chr23
5	RPS24	UTR3	ENSBTAG00000013264	chr28
6	COL6A3	exonic	ENSBTAG00000030190	chr3
7	RPL10	exonic	ENSBTAG00000007454	X
8	AIDA	UTR3	ENSBTAG00000007593	chr16
9	DNAJA1	exonic	ENSBTAG00000016265	chr8
10	NDE1	UTR3	ENSBTAG00000015986	chr25
11	TMA7	UTR3	ENSBTAG00000053755	chr22
12	SELENOP	UTR3	ENSBTAG00000054085	chr20
13	ENSBTAG00000043974	UTR3	ENSBTAG00000043974	chr25
14	QRFPR	UTR3	ENSBTAG00000008255	chr6
15	PKDCC	UTR3	ENSBTAG00000019382	chr11
16	CLU	UTR3	ENSBTAG00000005574	chr8
17	PRMT6	UTR3	ENSBTAG00000039951	chr3
18	CCDC152	UTR3	ENSBTAG00000033221	chr20
19	ENSBTAG00000048782	UTR5	ENSBTAG00000048782	chr10
20	RPL5	UTR3	ENSBTAG00000002026	chr3
21	NOTCH2	exonic	ENSBTAG00000007909	chr3
22	RRAGA	exonic	ENSBTAG00000033543	chr8
23	APEX1	UTR5	ENSBTAG00000002745	chr10
24	UQCR10	UTR3	ENSBTAG00000000913	chr17
25	ENSBTAG00000039890	exonic	ENSBTAG00000039890	X
26	ENSBTAG00000043974	exonic	ENSBTAG00000043974	chr25
27	KANK2	UTR3	ENSBTAG00000009568	chr7
28	MRPS33	UTR3	ENSBTAG00000005285	chr4
29	VASH1	exonic	ENSBTAG00000008735	chr10
30	ERBB2	UTR5	ENSBTAG00000021798	chr19
31	LSM7	UTR5	ENSBTAG00000004521	chr7
32	KDM4A	exonic	ENSBTAG00000002078	chr3
33	SHF	UTR3	ENSBTAG00000020934	chr10
34	TIMM8B	UTR3	ENSBTAG00000002000	chr15
35	TOP2B	exonic	ENSBTAG00000004593	chr27
36	SEC61A1	exonic	ENSBTAG00000004937	chr22
37	BCNT2	exonic	ENSBTAG00000030600	chr18
38	CDC42BPB	UTR3	ENSBTAG00000016456	chr21
39	SNRPD2	UTR5	ENSBTAG00000012177	chr18
40	ENSBTAG00000051573	exonic	ENSBTAG00000051573	chr25

Supplementary Table S7. The differential analysis data of m6A modification of BMP15

	m6A regulation INFO			
geneName	peak_annotation	P value	FC	m6A_regulation
BMP15	exonic	0.01	2.15	down

Supplementary Table S8. Crossbred cow weight and age

Group	Birth weight/kg	3 months old/kg	6 months old/kg	12 months old/kg
pre-1	24	65	150	
pre-2	30	70	140	
pre-3	21	65	155	
pre-4	24	70	155	
post-1	21	75	175	238
post-2	30	70	165	219
post-3	21	66.5	155	225
post-4	20	65	145	250

Supplementary Table S9. Sequence of transfection fragments

Gene	sense（3'→5'）	antisense（5'→3'）
BMP15-Bos-633	CCUUCUGAUUGAGGCAAAUTT	AUUUGCCUCAAUCAGAAGGTT
ALKBH5-Bos-32	GGGAGAAGCUCAAGUCCAUTT	AUGGACUUGAGCUUCUCCCTT
YTHDF2-Bos-1306	GGAUCUGGAUCUACUCCUUTT	AAGGAGUAGAUCCAGAUCCTT

Supplementary Table S10. Primer sequences of RT-qPCR

Classify	Gene	Primer sequences (5'→3')	Tm(℃)
hypothalamus	*MSC*	F: AAGCAGTCTCAGAGGAACGC	57
		R: TCGAAAGCTTGGTATCGGGG	
	KL	F: TCATCAAGGGAACAGCCGAC	57
		R: GGAGATTCCAGTTGGCGGAA	
	RGS14	F: TGAGCTGGTGTTTCAGGATGT	57
		R: GATGACGGGCAGATGACAGA	
pituitary	*VCAN*	F: TGCAGAAACTGTCTCACCCA	57
		R: GTGGCAGTGATTCTGTGGCT	
	PAPPA2	F: CGGAAGTTCACAGACACGGA	57
		R: AGCAGCCGTCTCCATTCAAA	
	IGSF10	F: AGGGGTGCCTGAGCTAGATT	57
		R: TGCAGGCTGACTTTTCCTCTT	
ovarian	*BMP15*	F: ATCATGCCATCATCCAGAACCTTGTC	62
		R: AGATACTCCCATTTGCCTCAATCAGAAG	
m6A	*METTL3*	F: AGCAGGACTCCACCACAGCAG	63
		R: CTTGGTTGGCTCCTTGGCTACTTC	
	METTL14	F: TGCCCCGAATTCCTATTTGACTGG	63
		R: CACCTCTTCCGCCTCCTCTGG	
	FTO	F: CTGCCAGCGTGCCTGAGTTG	63
		R: GTACCGTGAAGAGCCTGGTGTTC	
	ALKBH5	F: TGTCCTTCCAGGCCTAGTGT	61
		R: CCCTTCCTAGGGGGATGACA	
Cell proliferation	*cyclin D2*	F: CACCGATGTGGATTGCCTCA	58
		R: TCCAGCTCATCCTCCGACTT	
	cyclin A2	F: AGCAGCCTTTCATTTAGCACTCTAC	60
		R: GCTCTGAGGTAGGTCTGGTGAAG	

Continued

Classify	Gene	Primer sequences (5'→3')	Tm(℃)
Hormone synthesis	*CYP11A1*	F: CTACCAGGACCTGAGACGGA	58
		R: CCTGCCAGCATCTCCGTAAT	
	3β-HSD	F: TGCCACAATCTGACCGCATC	58
		R: CTCCACCAACAGGCAGATGA	
/	*β-actin*	F: TTGATCTTCATTGTGCTGGGTG	/
		R: CTTCCTGGGCATGGAATCCT	

Supplementary Table S11. Primer sequences of RT-qPCR

Gene	Primer sequences (5'→3')	Tm(℃)
BMP15	F: AGCCTTCCCTGTTGCCCAAAG	58
	R: AACTCACGAACCTCACTACCTCTTG	
